# Pharmacokinetics and clinical efficacy of midazolam in children with severe malaria and convulsions

**DOI:** 10.1111/j.1365-2125.2008.03239.x

**Published:** 2008-07-25

**Authors:** Simon N Muchohi, Gilbert O Kokwaro, Bernhards R Ogutu, Geoffrey Edwards, Steve A Ward, Charles R J C Newton

**Affiliations:** 1 Kenya Medical Research Institute (KEMRI)/Wellcome Trust Research Programme, Centre for Geographic Medicine Research (Coast) P.O. Box 230, 80108-Kilifi, Kenya; 2 Department of Pharmaceutics and Pharmacy Practice, School of Pharmacy, University of Nairobi Nairobi, Kenya; 3 Centre for Clinical Research, Kenya Medical Research Institute, Nairobi, Kenya/United States Army Medical Research Unit–Kenya (USAMRU–K) Kisumu, Kenya; 4 Department of Pharmacology and Therapeutics, School of Biomedical Sciences, University of Liverpool Liverpool L69 3GE, UK; 5 Molecular and Biochemical Parasitology Research Group, Liverpool School of Tropical Medicine Pembroke Place, Liverpool L3 5QA, UK; 6 Neurosciences Unit, The Wolfson Centre, Institute of Child Health, University College London London, UK; 7 London School of Hygiene and Tropical Medicine London, UK

**Keywords:** children, convulsions, malaria, midazolam, pharmacokinetics

## Abstract

**AIM:**

To investigate the pharmacokinetics and clinical efficacy of intravenous (IV), intramuscular (IM) and buccal midazolam (MDZ) in children with severe falciparum malaria and convulsions.

**METHODS:**

Thirty-three children with severe malaria and convulsions lasting ≥5 min were given a single dose of MDZ (0.3 mg kg^−1^) IV (*n* = 13), IM (*n* = 12) or via the buccal route (*n* = 8). Blood samples were collected over 6 h post-dose for determination of plasma MDZ and 1′-hydroxymidazolam concentrations. Plasma concentration–time data were fitted using pharmacokinetic models.

**RESULTS:**

Median (range) MDZ *C_max_* of 481 (258–616), 253 (96–696) and 186 (64–394) ng ml^−1^ were attained within a median (range) *t_max_* of 10 (5–15), 15 (5–60) and 10 (5–40) min, following IV, IM and buccal administration, respectively. Mean (95% confidence interval) of the pharmacokinetic parameters were: AUC(0,∞) 596 (327, 865), 608 (353, 864) and 518 (294, 741) ng ml^−1^ h; *V*_d_ 0.85 l kg^−1^; clearance 14.4 ml min^−1^ kg^−1^, elimination half-life 1.22 (0.65, 1.8) h, respectively. A single dose of MDZ terminated convulsions in all (100%), 9/12 (75%) and 5/8 (63%) children following IV, IM and buccal administration. Four children (one in the IV, one in the IM and two in the buccal groups) had respiratory depression.

**CONCLUSIONS:**

Administration of MDZ at the currently recommended dose resulted in rapid achievement of therapeutic MDZ concentrations. Although IM and buccal administration of MDZ may be more practical in peripheral healthcare facilities, the efficacy appears to be poorer at the dose used, and a different dosage regimen might improve the efficacy.

**WHAT IS ALREADY KNOWN ABOUT THIS SUBJECT**

- Midazolam (MDZ), a water-soluble benzodiazepine, can be administered via several routes, including intravenously (IV), intramuscularly (IM) and buccal routes to terminate convulsions. It may be a suitable alternative to diazepam to stop convulsions in children with severe malaria, especially at peripheral healthcare facilities. The pharmacokinetics of MDZ have not been described in African children, in whom factors such as the aetiology and nutritional status may influence the pharmacokinetics.

**WHAT THIS STUDY ADDS**

- Administration of MDZ (IV, IM, or buccal) at the currently recommended dose (0.3 mg kg^−1^) resulted in rapid achievement of median maximum plasma concentrations of MDZ within the range 64–616 ng ml^−1^, with few clinically significant cardio-respiratory effects. A single dose of MDZ rapidly terminated (within 10 min) seizures in all (100%), 9/12 (75%) and 5/8 (63%) children following IV, IM and buccal administration, respectively. Although IM and buccal MDZ may be the preferred treatment for children in the pre-hospital settings the efficacy appears to be poorer.

## Introduction

Acute convulsions are common in children admitted to hospitals in resource-poor countries, with over a fifth of children having a history of convulsions in their presenting illness in some areas [1]. In malaria-endemic areas, falciparum malaria is the most common cause of convulsions [2]. Multiple and prolonged convulsions are often refractory to treatment, and are associated with increased mortality [3, 4] and neurocognitive deficits in some survivors [5, 6]. Early treatment of convulsions may improve outcome in this group. The ideal anticonvulsant should be easy to administer, provide a rapid and sustained duration of anticonvulsant action, and be safe [7]. Benzodiazepines are often used as the drugs of choice for rapid termination of convulsions [8].

Diazepam is commonly used as the standard first-line treatment for acute convulsions in resource-poor countries, because it is inexpensive, rapidly acting, and widely available. However, it has several disadvantages. Diazepam is routinely administered IV, but this is not a practical route in many peripheral healthcare facilities in many parts of Africa. Intravenous administration of diazepam has a rapid onset of action, but it is rapidly redistributed into fatty tissues, leading to recurrence of convulsions [9]. Moreover, administration of multiple doses of diazepam is undesirable, especially in children with severe malaria, since respiratory depression may interfere with the hyperpnoea that compensates for the acidosis [10, 11]. In many parts of the world, the parenteral preparation of diazepam is administered rectally, but is erratically absorbed from this route [9]. Furthermore, it is unacceptable in some cultures. The IM route is commonly used in many peripheral healthcare units in Africa, but diazepam is slowly and erratically absorbed [12].

Midazolam (MDZ) has several pharmacokinetic and pharmacological properties that support its use. It can be administered via several routes, including IV [13–15], IM [16–19] and via the buccal cavity [20–24]. It is effective in childhood status epilepticus refractory to other anticonvulsants (diazepam, lorazepam, phenytoin and phenobarbital) [25, 26]. It has a rapid onset of action and shorter elimination half-life; it is therefore less likely than diazepam to accumulate and cause respiratory depression on repeated administration [14]. However, there are a number of issues that may limit the use of MDZ in severe malaria. Firstly, it is associated with decreased peripheral perfusion, which may limit absorption of IM administered drugs. Secondly, midazolam may impair the compensatory hyperpnoea and aggravate hypotension [27]. Lastly, the pharmacokinetics of MDZ may be influenced by hepatic dysfunction [28], antimalarials e.g. quinine which inhibits the metabolism of MDZ *in vitro*[29] and the plasma from patients with severe malaria [30].

The pharmacokinetics of MDZ have not been described in African children, particularly those with severe malaria. Moreover, the therapeutic concentration of MDZ has not been defined [31]. The objectives of this study were to describe the pharmacokinetics and clinical efficacy and safety of MDZ following IV, IM or buccal administration for treatment of Kenyan children with severe malaria and acute convulsions.

## Methods

### Study site and study design

This was an open-label, non-randomized, uncontrolled study. Children were recruited per route of drug administration (IV, IM and buccal, respectively) until the minimum number (target *n* = 12) had completed the full blood sampling procedure for the pharmacokinetic study. The study was conducted after approval by the Kenya Medical Research Institute (KEMRI)/National Ethical Review Committee and the Research Ethics Committee (Liverpool School of Tropical Medicine, Liverpool, UK). The study was conducted at the Kilifi District Hospital, located in a malaria endemic area on the Kenyan coast. A KEMRI research centre is situated within the hospital. Severely ill children who present to the hospital are assessed in the outpatient department by government-employed clinical officers. Children with severe malaria are admitted to the six-bed paediatric high dependency unit.

### Study participants

All children admitted to the high dependency unit were eligible for the study if they: (i) were aged between 6 months and 13 years; (ii) had features of severe malaria (blood-film positive *Plasmodium falciparum* plus one or more of the following: prostration (inability to sit or breast feed), coma, prolonged or recurrent convulsions, respiratory distress (deep breathing or intercostal recession), circulatory collapse, severe anaemia (haemoglobin <50 g l^−1^ plus respiratory distress) or jaundice) [32, 33]; (iii) had a convulsion lasting ≥5 min, or ≥3 convulsions lasting <5 min within 1 h; and (iv) if the child's parent(s)/guardian(s) gave written informed consent. Children were excluded if: (i) informed consent could not be obtained (they were treated with IV diazepam or IM paraldehyde); (ii) they had received diazepam before admission/recruitment into the study; (iii) they had compromised cardio-respiratory function (which might be worsened by administration of a benzodiazepine); and (iv) they had detectable MDZ concentrations in the pre-dose blood sample (excluded at the data analysis stage). Children exited the study if informed consent was withdrawn.

### Clinical care

Intravenous access was obtained by fixing F.E.P. polymer cannulae into the forearm veins, one for IV administration of fluids and drugs, and the other (in the opposite arm) for blood sampling. A venous blood sample (6 ml) was withdrawn for standardized investigations on admission including: quantitative malaria parasite count, blood culture, measurement of blood glucose, venous blood gases, electrolytes (sodium and potassium), lactate, and full blood count. Plasma from a portion of the blood (0.5 ml) was stored (−80°C) until analyzed for baseline MDZ and 1′-hydroxymidazolam concentrations.

Hypotension (defined as a systolic blood pressure <70 mm Hg in children aged <1 year, or <80 mm Hg in children aged >1 year) was treated by administering boluses of normal saline (20 ml kg^−1^ over 15 min) until the blood pressure was normal. Standard care was provided as previously described [9]. All convulsions were recorded on a proforma (type, duration, lowest oxygen saturation and blood glucose during the convulsion), with a 5-min post-ictal evaluation of the level of consciousness, respiratory rate and oxygen saturation. The nursing staff performed clinical assessments every 4–6 h, documenting vital signs, recurrence of convulsions and level of consciousness. Physicians reassessed the children at 4 and 24 h after admission and on a daily basis thereafter.

Malaria was treated with parenteral quinine (Ipca Laboratories Ltd, Kandivli Estate, Mumbai, India) (loading dose, 15 mg kg^−1^; maintenance dose: 10 mg kg^−1^ 12 hourly) diluted in 10 ml kg^−1^ of 5% dextrose) infused over 2 h [34] until patients could tolerate oral medication, when antimalarial therapy was completed with a full course of sulfadoxine–pyrimethamine alone or in combination with amodiaquine (according to Kenyan national guidelines at the time of the study). Antimalarial treatment was stopped if three consecutive blood smears at 8-hourly intervals were all negative.

Broad-spectrum antibiotics (chloramphenicol sodium succinate: loading dose of 40 mg base kg^−1^ plus maintenance dose of 25 mg base kg^−1^ 6 hourly; and benzyl penicillin: 60 mg base kg^−1^ 6 hourly) [35, 36] were given to all children as presumptive treatment for bacteraemia. This antibiotic regimen was continued until CSF and blood culture results were available.

Children with convulsions lasting ≥5 min were treated with a single dose (0.3 mg kg^−1^) of MDZ (Dormicum®; 15 mg per 3 ml; F. Hoffmann–La Roche Ltd, Basel, Switzerland), administered IV as a slow bolus over 1–2 min (to reduce the chances of respiratory depression), or as an IM injection into the anterior aspect of the thigh, or via the buccal cavity. For buccal administration, the appropriate volume (range: 0.3–1.0 ml) of the IV preparation of MDZ (15 mg per 3 ml) was drawn into a 1 ml syringe. The child's lips were parted and the MDZ solution squirted around the buccal mucosa between the gum and cheeks [23]. If the child had copious amounts of secretions following drug administration (often due to the ongoing convulsions), the nursing personnel had the discretion to commence suction to clear the airway (to avoid the risk of aspiration).

If the child was still having a convulsion 5–10 min after administration of MDZ, then IM paraldehyde (0.4 ml kg^−1^; 100% v/v, Mayne Pharma Plc, Warwickshire, UK) was administered. If the convulsions continued following administration of the first-line drugs (MDZ or paraldehyde), then phenytoin (Faulding Pharmaceuticals Plc., UK) was administered at a loading dose (18 mg kg^−1^) as an IV infusion over 20 min, followed by a maintenance dose (2.5 mg kg^−1^ 12 hourly) [37]. At 30 min, if the convulsions still persisted, phenobarbital (100 mg ml^−1^; Lab RENAUDIN, France) was administered IV as a loading dose (15 mg kg^−1^) infused over 20 min, followed by maintenance doses (2.5 mg kg^−1^) at 24 and 48 h [38].

Children with recurrent (≥3) convulsions lasting <5 min or status epilepticus (continuous clinical seizure activity lasting ≥30 min) were also treated with a loading dose of IV phenobarbital. Children who failed to respond to phenobarbital and were still convulsing at 60 min from start of treatment (refractory status epilepticus) were given sodium thiopental (500 mg, Biocheme, GmbH, Vienna, Austria) as an IV bolus (4 mg kg^−1^) infused over 1 min, followed by a maintenance dose of 4 mg kg^−1^ h^−1^ infused over 2 h under close supervision of a clinician.

### Blood sampling

Serial venous blood samples (0.5 ml) for the determination of plasma MDZ and 1′-hydroxymidazolam concentrations were collected before MDZ administration (pre-dose) and at 5, 10, 15, 20, 30, 40, 60 min, and 2, 3, 4, 5 and 6 h after drug administration. The blood was collected into lithium-heparinized tubes and immediately centrifuged (1500 *g*; 5 min) at room temperature. Plasma was separated and transferred into polyvinyl tubes and stored at −80°C until analyzed for MDZ and 1′-hydroxymidazolam.

### Clinical measurements

The times of administration of MDZ and termination of convulsions were recorded. Vital signs (respiratory rate, pulse rate, arterial blood pressure), oxygen saturation and level of consciousness (Blantyre coma score) [28] were recorded at every blood sampling time point. The total number, duration, and pattern of convulsions within the 6-h sampling period were also recorded. Efficacy was assessed by termination of the convulsion, latency (time from MDZ injection to convulsion control), and time to next convulsion. In a few cases, patients received a second or third anticonvulsant (paraldehyde, phenytoin or phenobarbital) after the single dose of MDZ. Therefore, the duration of convulsion free period was reported descriptively as ‘no recurrence’.

The primary outcome measure, therapeutic success, was the cessation of visible signs of seizure activity within 10 min of administration of MDZ without respiratory depression and without another convulsion within 6 h. Respiratory depression was defined as a fall in oxygen saturation to less than 95% or decrease in respiratory effort sufficient to require assisted breathing via bag-and-mask after administration of MDZ. Children who had respiratory depression following administration of MDZ were given flumazenil, a benzodiazepine receptor binding site antagonist [39].

### Determination of midazolam and 1′-hydroxymidazolam by LC–MS–ESI

Plasma concentrations of MDZ and 1′-hydroxymidazolam were simultaneously determined using a sensitive and selective reversed-phase high-performance liquid chromatography method coupled with electrospray ionization mass spectrometry (LC–ESI–MS) [40]. Briefly, MDZ, 1′-hydroxymidazolam and 1′-chlordiazepoxide (internal standard) were extracted from alkalinized (pH 9.5) plasma samples using liquid–liquid extraction with 1-chlorobutane. Separation was performed on a reversed-phase HyPURITY™ Elite C_18_ analytical column (100 mm × 2.1 mm i.d.; 5 µm) by isocratic elution using an acidic (pH 2.8) mobile phase (water-acetonitrile; 75 : 25% (v/v) containing formic acid (0.1%, v/v)). Flow rate was 0.2 ml min^−1^. The mass spectrometer was operated in the positive ion mode at the protonated molecular ions [M+l]^+^ of parent drug and metabolite. Calibration curves were linear (*r* ≥ 0.995) over the plasma concentration range of 15–600 ng ml^−1^ (MDZ) and 5–200 ng ml^−1^ (1′-hydroxymidazolam). The limits of detection and quantification were 2 ng ml^−1^ and 5 ng ml^−1^, respectively, for both MDZ and 1′-hydroxymidazolam. The intra-assay coefficients of variation (CVs) at 40, 250 and 500 ng ml^−1^ of MDZ were 4.4%, 6.5% and 1.3% (*n* = 7 in all cases), respectively; the CV values for 1'-hydroxymidazolam at 30, 80 and 180 ng ml^−1^ were 2.4%, 9.7% and 4.9% (*n* = 7 in all cases), respectively. The inter-assay CVs at the above concentrations were 6.9%, 4.1% (*n* = 7 in both cases) and 6.5% (*n* = 8), respectively, for MDZ and 12.8%, 7.5% (*n* = 7 in both cases) and 5.0% (*n* = 8), respectively, for 1'-hydroxymidazolam. There was no interference from other commonly co-administered (anticonvulsant, antimicrobial, antipyretic, and antimalarial) drugs in these children. 4′-Hydroxymidazolam did not interfere with the assay, but it was not detected in all the patient samples.

### Pharmacokinetic analysis

Plasma MDZ and 1'-hydroxymidazolam concentration–time data after IV, IM and buccal administration were used to estimate standard pharmacokinetic parameters (clearance, elimination half-life, apparent volume of distribution and area under the plasma drug concentration–time curve) using the pharmacokinetic program TopFit (Version 2.0) [41] and standard pharmacokinetic methods [42]. The maximum plasma drug concentration (*C_max_*) and the corresponding time to achieve this value (*t_max_*) were noted directly.

### Statistical analysis

The estimated pharmacokinetic parameters were summarized as mean or median (95% confidence interval (95% CI)) or median (range). The 95% CI for the differences between the means or medians were calculated using a commercially available statistical program Confidence Interval Analysis (CIA) [43]. The Mann–Whitney U-test was used to compare demographic and pharmacokinetic parameters between the IV, IM and buccal treatment groups, using Stata*™* (version 9.0) (Stata Corporation, Texas, USA). A *P* value of <0.05 was considered statistically significant.

## Results

### Demographic characteristics

Thirty-three children (median age, 26 months; range: 7–86 months) were recruited into the study (Table 1). There were no statistically significant differences in the demographic and laboratory parameters on admission among the study groups, and they were similar to most children admitted with severe malaria in this population [9].

**Table 1:** Demographic characteristics and biochemical parameters of children administered a single dose (0.3 mg kg^-^^1^) of midazolam (MDZ) intravenously (i.v.), intramuscularly (i.m.) or via buccal cavity

Parameter	IV midazolam	IM midazolam	Buccal midazolam	*P* value
***Demography***				
**Number of children**	13	12	8	–
**Gender (M : F)**	5 : 8	5 : 7	4 : 4	–
Age (months)*	27 (7–39)	25 (9–96)	26 (7–64)	0.852
**Weight (kg)**	10.4 (8.7, 12.1)	11.3 (8.8, 13.9)	10.1 (7.9, 12.4)	0.872
**Weight-for-age Z (WAZ) score**	−1.6 (−3.6, 1.8)	−1.3 (−4.2, 0.0)	−1.6 (−4.0, −0.1)	
**Number of malnourished children (WAZ ≤3)**	3 (23%)	1 (8%)	2 (25%)	–
***Laboratory parameters***				
**Haemoglobin (g dl^−1^)**	7.3 (6.1, 8.5)	7.9 (6.2, 9.7)	7.6 (4.5, 10.7)	0.611
WBC (×10^6^); µl^−1^*	11.8 (8.2, 15.5)	15.6 (8.7, 22.4)	19.7 (6.2, 33.2)	0.321
**Parasite count (µl^−1^); geometric mean**	36 163	69 255	26 242	–
**Sodium (mmol l^−1^)**	138 (136, 140)	134 (130, 137)	133 (131, 135)	0.059
**Potassium (mmol l^−1^)**	4.4 (4.0, 4.7)	4.2 (3.8, 4.6)	3.9 (3.3, 4.6)	0.295
**Creatinine (µmol l^−1^)**	55 (31, 79)	53 (40, 66)	41 (32, 50)	0.198
**Blood glucose (mmol l^−1^)**	8.1 (4.4, 11.7)	6.7 (4.2, 9.2)	4.3 (−0.65, 8.3)	0.294
**Hypoglycaemia (blood glucose <2.2 mmol l^−1^); (*n*)**	1	1	4	–
**pH**	7.16 (6.98, 7.33)	7.25 (7.18, 7.32)	7.12 (6.96, 7.28)	0.854
**Base excess**	−10.5 (−14.9, −5.9)	−8.3 (−11.5, −5.2)	−12.3 (−15.2, −9.4)	0.852

Values are expressed as mean (95% confidence interval; CI) or

* median (range), except parasite count, which is expressed as geometric mean.

### Control of convulsions

A single dose of MDZ terminated convulsions in all (13/13; 100%), 9/12 (75%) and 5/8 (63%) children, following IV, IM, and buccal administration, respectively. These convulsions were terminated within a median time of 10 min (Table 2), otherwise other antiepileptic drugs were used. Two children had convulsions that were refractory to all the anticonvulsants used. The therapeutic success rate (defined as cessation of visible signs of seizure activity within 10 min of administration of MDZ without respiratory depression and without another convulsion within 6 h) was 46%, 50% and 40%, respectively, for the IV, IM and buccal routes, respectively.

**Table 2:** Clinical progress and outcome

	IV midazolam	IM midazolam	Buccal midazolam
**Number of children**	13	12	8
**Blantyre coma score on admission**	3 (0–5)	2 (0–5)	3 (0–5)
**Blood transfusion (*n*)**	1 (8%)	2 (17%)	0 (0%)
**Number of convulsions after admission**	3 (1–uncountable)	2 (1–uncountable)	1 (1–3)
**Patients with convulsions controlled with a single dose of midazolam**	13 (100%)	9 (75%)	5 (63%)
**Latency (time from initial midazolam administration to convulsion control) (min)**	2 (1–6)	5 (1–5)	3.5 (1–5)
Patients with recurrence convulsions after midazolam*	6 (46%)	2 (22%)	0 (0%)
**Patients requiring additional anticonvulsants:**			
** Paraldehyde**	3 (23%)	1 (8%)	0 (0%)
** Phenobarbital**	4 (31%)	4 (33%)	3 (38%)
** Phenytoin**	3 (23%)	3 (25%)	3 (38%)
** Thiopental**	1 (8%)	0 (0%)	0 (0%)
**Patients with respiratory depression after midazolam**	1 (8%)	1 (8%)	2 (25%)*
**Deaths**	0 (0%)	1 (8%)	3 (38%)

Values are expressed as number (percentage) or number (range).

* Statistically significantly different from the IV MDZ group (*P* = 0.029).

### Cardio-respiratory effects

Four children (one in the IV, one in the IM and two in the buccal groups) (12%) had respiratory depression immediately after MDZ administration. Flumazenil successfully reversed the respiratory depressant effects of MDZ.

### Deaths

Four children (one in the IM group and three in the buccal group) died. However, none of these children died during the 6-h sampling period. All these children had cerebral malaria and multiple convulsions, three had hypoglycaemia on admission, and in addition, one child had bacterial meningitis. The deaths were attributed to complications associated with severe falciparum malaria such as severe metabolic acidosis and hypoglycaemia.

### Pharmacokinetics

**Figure 1:**
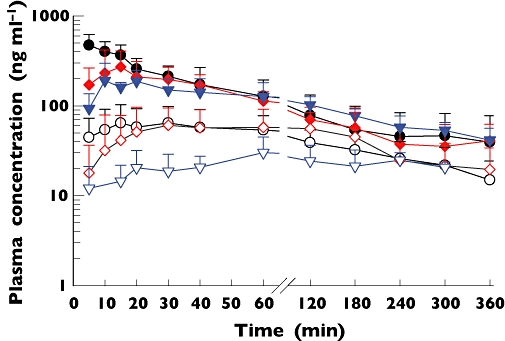
Mean (SD) plasma concentration *vs.* time profiles of midazolam (MDZ) and 1′-hydroxymidazolam following administration of a single dose (0.3 mg kg^−1^) of midazolam intravenously (*n* = 12; MDZ; •; 1′-hydroxymidazolam: ○), intramuscularly (*n* = 11; MDZ: 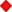, 1′-hydroxymidazolam: 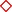) or via the buccal cavity (*n* = 8; MDZ: 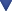, 1′-hydroxymidazolam: 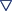) to children with severe malaria and convulsions

The pharmacokinetic profiles of 1′-hydroxymidazolam paralleled those of the parent compound (Figure 1). The ratios of AUC(0,∞) of 1′-hydroxymidazolam to parent drug were 0.49–0.55 for the three routes of administration (Table 3). 4′-Hydroxymidazolam concentrations were below the limit of quantification in all the children who received midazolam by IV, IM or buccal routes.

**Table 3:** Pharmacokinetic parameters of midazolam (MDZ) and 1′-hydroxymidazolam following administration of a single dose (0.3 mg kg^−1^) of MDZ intravenously (IV), intramuscularly (IM) or via the buccal cavity in children with severe malaria and convulsions

Parameter	IV midazolam	IM midazolam	Buccal midazolam	95% CI for the difference between means or ‡medians		
				IV *vs.* IM	IV *vs.* buccal	IM *vs.* buccal
**Number of children**	12	11	8			
***Midazolam***						
***C_max_* (ng ml^−1^)‡**	481 (358, 554)	253 (145, 475)	186 (64, 394)	79, 322*	135, 369*	−37, 220
***t_max_* (h)‡**	0.167 (0.08, 0.167)	0.25 (0.167, 0.67)	0.27 (0.12, 0.50)	0.0, 0.33	−0.25, 0.0	−0.16, 0.25
***t_1/2_* (elimination) (h)**	1.22 (0.65, 1.8)	–	–			
**AUC (0,∞) (ng ml^−1^ h)**	596 (327, 865)	608 (353, 864)	518 (294, 741)	−366, 340	−433, 278	−413, 233
**CL (ml^−1^ min^−1^ kg^−1^)**	14.4 (9.2, 19.7)	–	–			
***V*_d_ (l kg^−1^)**	0.85 (0.49, 1.13)	–	–			
**Bioavailability (*F*)**	Assume 100%	102%	87%			
1′-Hydoxymidazolam**						
***C_max_* (ng ml^−1^)‡**	64 (44, 103)	82 (18, 116)	46 (16, 102)	−29, 42	−56, 20	−24, 66
***t_max_* (h)‡**	0.59 (0.167, 1.0)	0.67 (0.5, 2.0)	1.0 (0.5, 2.0)	−0.67, 0.33	−0.33, 0.83	−0.33, 0.50
**AUC(0,∞) (ng ml^−1^ h)**	197 (118, 276)	270 (151, 389)	189 (61, 317)	−56, 203	−139, 123	−87, 249
**AUC_*1–hydroxymidazolam*_ : AUC_*midazolam*_**	0.55 (0.11, 0.99)	0.49 (0.27, 0.72)	0.55 (0.07, 1.04)	−0.44, 0.55	−0.63, 0.64	0.37, 0.49*

Values are presented as mean (95% confidence interval, CI) or

‡ median (95% CI).

* Statistically significantly different from the IV MDZ group.

** *n* = 6 for buccal MDZ; calculation of pharmacokinetic parameters were possible in only these number of cases.

**Figure 2:**
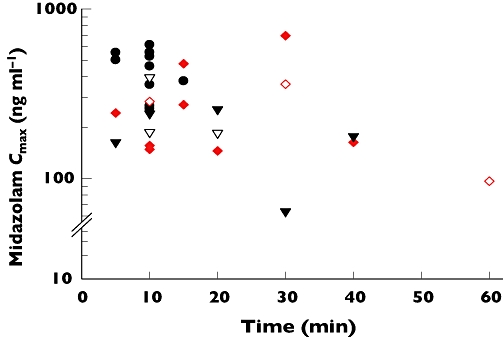
Relationship between midazolam (MDZ) plasma maximum concentrations (*C_max_*) and *t_max_* and efficacy in termination of convulsions in children following administration of a single dose (0.3 mg kg^−1^) of MDZ intravenously (IV) (•: seizures terminated, *n* = 12); intramuscularly (IM) (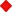: seizures terminated, *n* = 8; 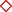: seizures not terminated, *n* = 3); or via the buccal route (▾: seizures terminated, *n* = 5; ▿: seizures not terminated, *n* = 3)

Midazolam was rapidly absorbed after IM and buccal administration. Median plasma MDZ *C_max_* values within the range 64–616 ng ml^−1^ were achieved within 15 (range 5–60) min in all the children, for the three routes of administration (Figure 1 and Table 3). Compared with the IV route, *C_max_* values were statistically lower following IM and buccal administration, but the values were not statistically significantly different between IM and buccal administration (Table 3). The *C_max_* values (range 96–360 ng ml^−1^ for IM and 185–394 ng ml^−1^ for the buccal route) for those children whose convulsions were not terminated were within the 95% CI for the group (Figure 2).

Six (18%) children (three in the IV, one in the IM and two in buccal groups, respectively) were malnourished (weight-for-age Z (WAZ) score ≤−3.0; range −3.1 to −4.2) (Table 1). The *C_max_* (96 ng ml^−1^) and *t_max_* (1.0 h) for the malnourished child in the IM group were outside the 95% CI for this group, but the MDZ *C_max_* values for IV (range 258–268 ng ml^−1^) and buccal (range 163–187 ng ml^−1^) routes, and the corresponding *t_max_* values (range 0.08–0.167 h), respectively, were within the 95% CI for the two groups.

## Discussion

This study shows that MDZ administered IV is more effective in the termination of convulsions than via the IM and buccal routes in children within a hospital setting, but it is associated with a risk of respiratory depression. Because of its good aqueous solubility, MDZ offers superior uptake and rapid absorption after IM [19] and buccal [44] administration. After an initial rapid fall in MDZ concentration characterized by the redistribution phase, the IV concentration–time plot showed a log-linear decline (elimination phase). The parallel decline in plasma concentrations of MDZ and 1′-hydroxymidazolam and the ratio of AUC_1-hydroxymidazolam_ : AUC_midazolam_ <1 (Table 3) are consistent with formation rate-limited metabolite kinetics, as reported in young healthy volunteers [45].

### Pharmacokinetics

The pharmacokinetics of MDZ in this study are similar to other studies in children and neonates [13, 17, 46]. The results from the present study show that the exposure (based on AUC) for the IM and buccal routes suggests almost complete bioavailability (Table 3). In comparison, the absolute bioavailability for IM MDZ was approximately 87% in children aged 5.9 years [17] and 87 ± 18% in adult epileptic patients [16]. The high bioavailability after IM and buccal administration of MDZ supports its administration by this route.

4′-Hydroxymidazolam concentrations were below the limit of quantification in all the children who received MDZ by IV, IM or buccal routes. In 10 critically ill adult patients with acute renal failure on continuous venovenous haemofiltration, the amounts of 4′-hydroxymidazolam and 4′-hydroxymidazolam-glucuronide excreted were less than 1% of the amounts of 1′-hydroxymidazolam and 1′-hydroxymidazolam-glucuronide [47]. Therefore, it is likely that in the children in our study, the 4′-hydroxymidazolam metabolite was formed in very low quantities, and subsequently rapidly conjugated and eliminated in urine. 1'-Hydroxymidazolam is as potent as the parent drug [48]; it may contribute to the pharmacological activity of the drug following oral administration.

Two children had copious amounts of saliva following administration of buccal MDZ, necessitating suction of the airway. MDZ *C_max_* values of 161 and 63 ng ml^−1^ were achieved within 5 and 30 min, respectively; these values were within the 95% CI for the group. However, convulsions were terminated in both cases. None of the children had any aspiration.

The pharmacokinetic parameters of MDZ are reported to exhibit a considerable degree of inter-individual variability, particularly in critically ill patients [49]. There was approximately a two-fold variation in plasma MDZ clearance in patients in this study. Hughes *et al.*[50] reported a higher plasma MDZ clearance (13 ml^−1^min^−1^ kg^−1^) in children aged ≥3 years compared with infants and children up to 2 years of age (3.1 ml^−1^min^−1^ kg^−1^ and 2.3 ml^−1^min^−1^ kg^−1^, respectively).

### Efficacy

In the present study, the primary outcome measures included cessation of visible signs of convulsive activity within 10 min of drug administration, without respiratory depression requiring medical intervention, and without another convulsion within the 6-h study period. Following administration of buccal MDZ, convulsions were terminated within 10 min in five out of eight children (63%), which is in accord with values (75–78%) reported in previous studies [20, 21, 23]. Buccal MDZ is rapidly effective [44] and in previous studies, buccal administration terminated convulsions within 3–20 min of administration in 56–84% of cases [20, 22–24]. Buccal MDZ was shown to be at least as effective as rectal diazepam in the acute treatment of seizures [21, 24]. In a recent study of Ugandan children [20], buccal MDZ was superior to rectal diazepam for treatment of children with prolonged convulsions, but the benefits of buccal MDZ were markedly evident in children with convulsions that were not associated with falciparum malaria.

Most of the children in the present study had recurrence of convulsions following initial termination with IV or IM MDZ (Table 2). The incidence of recurrence of convulsions after administration of MDZ has been previously reported: 11–31% for IM [19, 51], 14–30% for buccal [20, 22, 24] administration, and 57% for continuous MDZ IV infusion for the control of refractory status epilepticus [52]; this is attributed to the short elimination half-life (about 1–3 h) of MDZ. The relatively lower efficacy of buccal and IM administration compared with IV MDZ may be attributed to a lag in the absorption phase, especially following IM administration. The prolonged absorption phase may be caused by malaria which is associated with decreased peripheral perfusion. One malnourished child had lower C*_max_* and longer *t_max_* values compared with non-malnourished children. It is likely that MDZ was poorly absorbed from the IM injection site.

### Regimen

The doses of MDZ used in children in previous studies were variable, and there are few pharmacokinetic data to justify the dosage regimens. The dose of MDZ used in the present study (0.3 mg kg^−1^) is within the range (0.2–0.3 mg kg^−1^) used in previous studies in children [22–24], but lower than the buccal dose (0.5 mg kg^−1^) used in recent studies by Mpimbaza *et al*. [20] and McIntyre *et al.*[24]. In the present study, only a single dose of MDZ was given. Repeated administration of MDZ may result in more effective control of seizures. Accumulation of drug and its metabolites may not occur (because of the short elimination half-lives). The weight-adjusted dose of buccal MDZ that would be sufficient to control seizures in this group has not been defined, and it is still unclear whether efficacy and safety of this dose would be dependent on age.

### Potential drug interactions

The effect of other drugs on the pharmacokinetics of MDZ may be clinically significant. All the children in this study received quinine, which is metabolized to 3-hydroxyquinine by CYP3A4 [53] and has been shown to interact with MDZ *in vitro*[54]. However, it was not possible to measure plasma concentrations of quinine in this study because only limited blood volumes could be taken from these severely ill young children. MDZ has an intermediate to high extraction ratio of approximately 0.4 [55], and its clearance may vary with both hepatic blood flow and hepatic enzyme activity. The variability in the latter may be much more important, because MDZ is eliminated through oxidation by hepatic enzymes, with a restricted capacity.

### Adverse events

Four (12%) children in this study experienced respiratory depression, compared with 1.2% and 5% in recent studies [20, 24]. Respiratory depression associated with convulsions may probably be due to several factors, one of which is benzodiazepine therapy [56]. Four deaths (12%) occurred among the children who were enrolled in the present study. However, none of the children died during the 6-h study period; all the children died at least 24 h after MDZ administration. Thus, it is unlikely that MDZ was responsible for the deaths.

### Limitations of the study

The limitations of this study include: (i) a non-randomized study design which potentially may have introduced selection bias in the allocation of patients into the different routes of MDZ administration; and (ii) the small numbers in each group limits the comparison between the groups. Further studies are required to compare the efficacy and frequency of adverse events between the different routes of administration.

In conclusion, the pharmacokinetic data presented in this study show a high bioavailability and reliable plasma concentrations following IM and buccal MDZ. Administration of MDZ (IV, IM or buccal) at the currently recommended dose resulted in rapid attainment of maximum plasma concentrations of MDZ above 60 ng ml^−1^ but it was associated with a high incidence of respiratory depression. Although IV MDZ terminated seizures in all children with severe malaria, there was a high rate of recurrence of seizures. Intramuscular and buccal administration of MDZ may be more practical in most rural healthcare facilities in sub-Saharan Africa, but the efficacy appears to be poorer at the dose used, and a different dosage regimen might improve the efficacy of IM and buccal MDZ. We recommend that MDZ (0.3 mg kg^−1^) should be administered IV, but in peripheral health centres with no facilities for IV cannulation, then IM and buccal MDZ may be used cautiously as an alternative. Ideally, MDZ should be administered together with a long acting anticonvulsant in children with severe malaria and convulsions.

*Competing interests: The authors declare that they have no competing interests. The Wellcome Trust (UK) and WHO (TDR/MIM) financially supported this work. They had no role in the collection, analysis and interpretation of data or in the writing of the manuscript.*

*We would like to thank the medical, nursing, laboratory and other staff of the pediatric high-dependency unit at the Kenya Medical Research Institute for their dedication and valuable support. We are indebted to the children and their parents/guardians, for participating in this study. We thank Professor Kevin Marsh and Dr Norbert Peshu (KEMRI/Wellcome Trust Research Programme, Kilifi) for their support. Professor Gilbert O. Kokwaro was supported by a Research Capability Strengthening Grant from WHO (TDR/MIM grant nos. 980074 and A50071). Dr Simon Ndirangu Muchohi was a PhD student in clinical pharmacology supported by The Wellcome Trust of Great Britain. Professor Charles R.J.C. Newton is a Wellcome Trust Senior Clinical Research Fellow (grant no. 070114/Z/02/Z). This paper is published with permission from the Director of KEMRI.*
